# Ultrasound-guided bilateral modified-thoracoabdominal nerve block through a perichondrial approach (M-TAPA) in patients undergoing laparoscopic cholecystectomy: a randomized double-blind controlled trial

**DOI:** 10.1186/s12871-022-01866-4

**Published:** 2022-10-28

**Authors:** Ayşegül Bilge, Betül Başaran, Tayfun Et, Muhammet Korkusuz, Rafet Yarımoğlu, Hatice Toprak, Nuh Kumru

**Affiliations:** 1 Department of Anesthesiology and Reanimation, Karamanoglu Mehmetbey University, Karaman, Turkey; 2 Department of Anesthesiology and Reanimation, Karaman Training and Research Hospital, Karaman, Turkey; 3 Department of Anesthesiology and Reanimation, Faculty of Medicine, Karamanoglu Mehmetbey University, Universite Mh. Sehit Omer Halis Demir Street, No:7, Karaman, Turkey

**Keywords:** Analgesia, Laparoscopic cholecystectomy, Nerve block, Pain management, Postoperative pain, Ultrasonography

## Abstract

**Background:**

Modified thoracoabdominal nerve block through the perichondrial approach (M-TAPA) is a new technique that provides effective analgesia of the anterior and lateral thoracoabdominal walls by administering local anesthesia only to the underside of the perichondral surface. The primary purpose of the present study was to investigate the postoperative analgesic efficacy of M-TAPA block performed before surgery in patients undergoing laparoscopic cholecystectomy (LC).

**Method:**

The present study was designed as a double-blind, randomized, controlled, prospective study. A total of 68 patients were included in the study. In group M-TAPA, M-TAPA block was performed bilaterally *after the induction of general anesthesia*. No block was performed on the group control. The postoperative pain scores, analgesic use in the first 24 h, antiemetic consumption, sedation, postoperative nausea and vomiting (PONV), and Quality of Recovery-40 (QoR-40) scores were recorded.

**Results:**

Pain scores were significantly lower in group M-TAPA than in the group control, both during resting and motion at all times (p < 0.001 at each time point). The total amount of tramadol consumed in the first 24 h was lower in group M-TAPA [median 100 mg, min-max (0-200)] than in the group control (P < 0.001). Postoperative median QoR-40 scores were higher in group M-TAPA compared with the group control (P < 0.001). There were no differences between the groups in terms of other results.

**Conclusion:**

After the LC surgery, ultrasound-guided M-TAPA block reduced postoperative pain scores and tramadol consumption effectively. It was observed that the quality of recovery was also higher because QoR-40 scores were higher.

## Background

Laparoscopic cholecystectomy (LC) brings benefits such as less pain, shorter hospital stay, and earlier recovery, and it is a cost-effective procedure [1]. Although LC is a minimally invasive surgery, it causes moderate-severe pain [2]. Relief of this pain is an issue of great clinical importance. Pain has several sources. These are a combination of visceral and reflected shoulder pain with incisional pain, which affect patients the most [2].

Nonsteroidal anti-inflammatory drugs, paracetamol, opioids, local anesthetic (LA), and many different regional anesthesia techniques are used to reduce postoperative pain caused by LC [3]. Oblique subcostal transversus abdominis plane block (OSTAP), and serratus intercostal plane block (SIP) are regional anesthesia techniques defined for use in supraumbilical surgeries [4, 5]. However, they are insufficient alone in blocking both the lateral and anterior areas of the abdominal surface [4, 5]. Ultrasound-guided erector spinae plane (ESP) block is a plane block that was reported to provide effective analgesia after LC, blocking the dorsal and often ventral branches of spinal nerves [6]. However, ESP block cannot be performed in the supine position. The anterior abdominal wall is innervated by the upper (T6-9) and lower thoracoabdominal (T10-12) nerves [7]. Thoracoabdominal nerves through perichondrial approach (TAPA) block is a novel block affecting both anterior and lateral branches of the thoracoabdominal nerves [8].

M-TAPA block was defined by Tulgar et al. for postoperative analgesia in abdominal surgeries. In this modified technique, local anesthetic given to the lower surface of the chondrium provides a wide blockage area that includes T5 and T11-12 dermatomal levels because the applied LA also passes the linea semilunaris [9, 10]. M-TAPA block also has another advantage because it can be applied in the supine position. No randomized controlled clinical studies have shown the analgesic efficacy of M-TAPA block primarily using a control group in abdominal surgery.

The primary purpose of the present study was to investigate the analgesic efficacy of M-TAPA block performed before surgery in patients undergoing LC. Secondary objectives were evaluating tramadol use, the incidence of complications, and Quality of Recovery-40 (QoR-40) scores in the first 24 postoperative hours.

## Methods

### Study Design

**Fig. 1:**
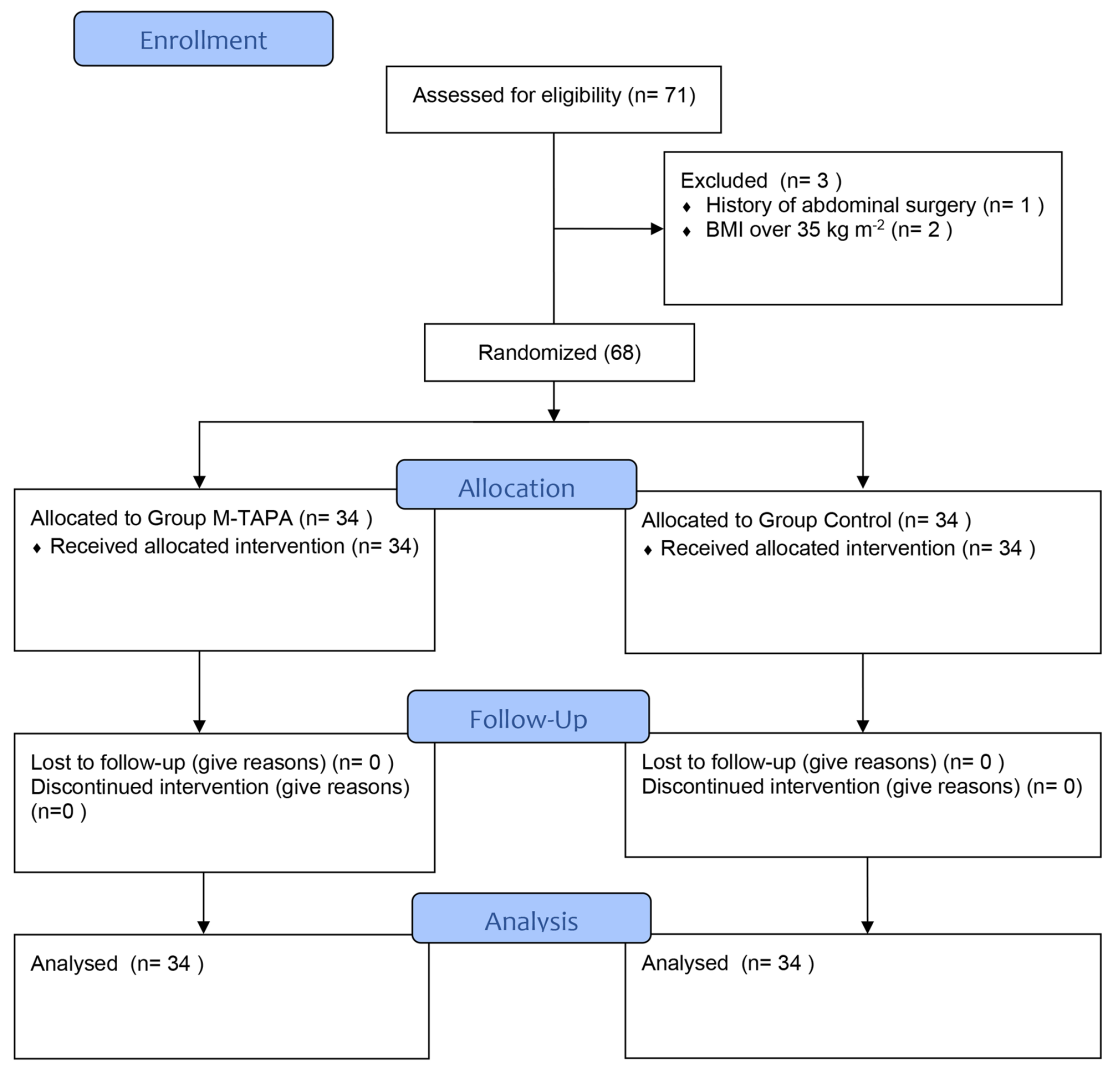
**CONSORT diagram of the study**

This prospective, double-blind, randomized controlled trial was conducted from August 24th to December 10th, 2021. Institutional Review Board approval (05-2021/09) of Karamanoglu Mehmetbey University Faculty of Medicine, Turkey was obtained on August 03, 2021. The study was designed in accordance with the principles set out in the Declaration of Helsinki and registered prospectively at clinicaltrials.gov (NCT05017090) on 23/08/2021. The Consolidated Standards of Reporting Trials (CONSORT) checklist was used for patient enrollment (Fig. 1). After providing written informed consent, patients aged 18–70 years with American Society Anesthesiologists (ASA) physical status classification scores of I or II scheduled for elective LC were included in the study. The exclusion criteria were the presence of coagulation disorder, infection at the injection site, known allergy to LA, advanced liver or kidney failure, history of abdominal surgery, or trauma, conversion to open surgery, use of any pain killers in the preoperative 24 h, chronic opioid consumption, alcohol or drugs use, refusal to participate, not being able to communicate in Turkish, pregnancy, and body mass index (BMI) ≥ 35 kg/m^2^.

### Anesthesia application

Standard monitoring with electrocardiography, non-invasive blood pressure, capnography, a multi-gas analyzer, and peripheral oxygen saturation was performed on all patients in the operating room. Mechanical ventilation was achieved using a pressure-controlled mode to maintain end-tidal carbon dioxide at 35 to 40 mm Hg. Depth of anesthesia was controlled during surgery using end-tidal sevoflurane and maintaining the sevoflurane concentration (0.8-1MAC). M-TAPA was administered bilaterally by a single anesthesiologist after the induction of general anesthesia before the surgical procedure.

Anesthesia induction and endotracheal intubation were performed intravenously using propofol 2 mg/kg, fentanyl 0.5-1 µg/kg, and rocuronium bromide 0.6-1 mg/kg. Anesthesia was maintained with sevoflurane and remifentanil infusion. Remifentanil was administered with an infusion dose of 0.01–0.1 mcg/kg/h, within the limits of 20% of the pre-anesthetic mean arterial blood pressure value.

The surgery was performed by the same single surgeon using the standard 4-trocar method. A total of four trocars were placed on the superior umbilicus, epigastric region under the xiphoid, and right midclavicular, right anterior axillary line within sub-costal area. The gas pressure was maintained at 12 mm Hg to create the pneumoperitoneum. Tramadol was administered at a dose of 1 mg/kg before discontinuing remifentanil at the end of surgery. The Aldreth score was > 9 in all patients who left the post-anesthesia care unit (PACU).

### Patient randomization and blinding

The patients were divided randomly into two groups according to the computerized randomization table that was created by a researcher who was not involved in the study. The operating room anesthetist retrieved the corresponding sealed envelope from a file for each randomized patient, indicating the treatment to be assigned to the patient. The anesthesiologist who was involved in block performance was not involved in postoperative data collection. The patients were also blinded to the group allocation; the blinding of the patients was provided by covering the M-TAPA block application sides with dressing in both groups.

### M-TAPA technique

**Fig. 2:**
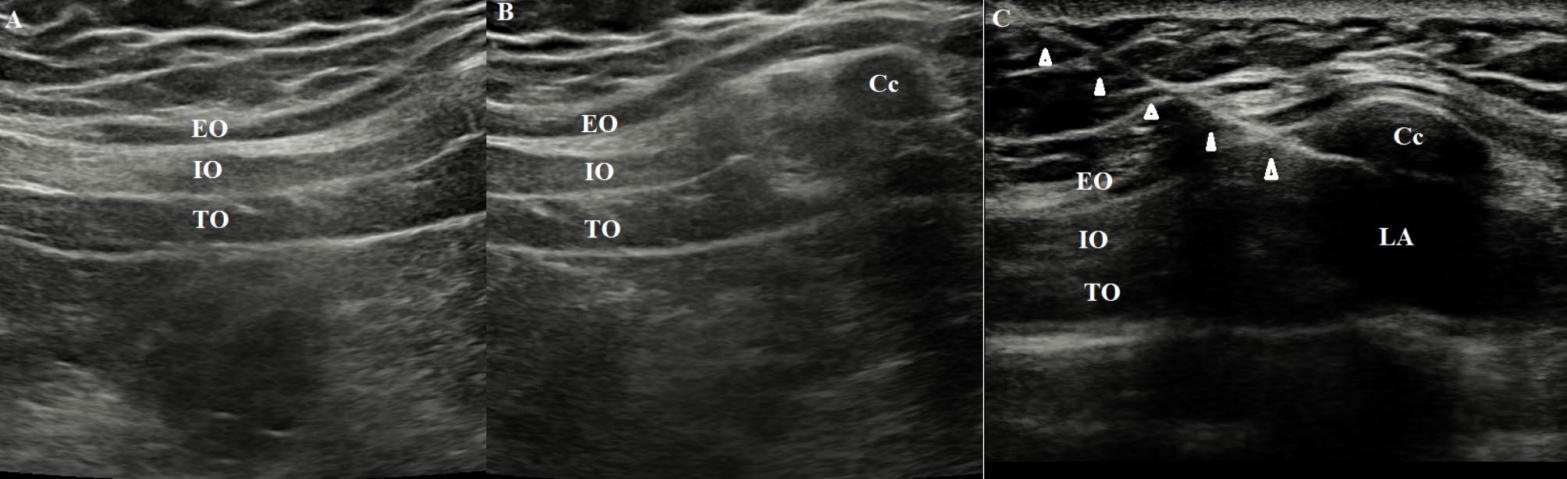
**Ultrasonographic view of process** (A) Ultrasonographic view of the abdominal muscles (B) Ultrasound image of the perichondral area before blocking (C) Sonographic view of the block needle and injection point at the lower aspect of chondrium. Cc: costal cartilage, EO: external oblique muscle, IO: internal oblique muscle, TO: transversus abdominis muscle, LA: local anesthetic

M-TAPA was performed bilaterally by a single anesthesiologist, as described by Tulgar et al. [9]. Following the tracheal intubation and before the surgical procedure, transversus abdominis, internal oblique, and external oblique muscles were identified with a high-frequency (12 MHz) linear probe on the costochondral angle in the sagittal plane under ultrasound guidance at the 10th costal margin. A deep angle was given to the costochondral angle at the edge of the 10th costa with the probe in the sagittal direction to view the lower surface of the costal cartilage in the midline. A 21-G, 80-mm block needle was inserted in the cranial direction using the in-plane technique and the needle tip was moved to the posterior aspect of the 10th costal cartilage. It was noted that the needle tip never crossed the cranial edge of the 10th costal cartilage and 25 mL of 0.25% bupivacaine was injected into the lower surface of the chondrium (Fig. 2). The same process was repeated for the other side.

### Outcome measures and data collection

The primary purpose of the study was to evaluate the numerical rating scale (NRS) score at rest and during movement in the first postoperative 24 h in patients with and without M-TAPA block. Pain severity was measured using an NRS:0, no pain; 10, worst pain imaginable. NRS scores were recorded postoperatively at minutes 0, 15, 30, and 60, and hours 2, 6, 12, and 24. Patients were educated and familiarized with NRS scores in the preoperative period.

Secondary endpoints were evaluating the preoperative and postoperative patient-completed QoR-40 questionnaire, sedation score, tramadol consumption at designated hours, the incidence of nausea and vomiting (PONV score), and consumption of antiemetics.

The QoR-40 questionnaire is a self-report questionnaire used to assess the quality of postoperative recovery and the health of patients in the early postoperative stages. Each item is rated on a scale of 1–5, reaching a minimum of 40 and a maximum of 200 points. In the QoR-40 questionnaire, a score of 40 represents the lowest recovery, and 200 represents an extraordinary quality of recovery. All patients were asked to complete the QoR-40 questionnaire twice, on the morning of the surgery in the preoperative waiting area, and 24 h after the surgery.

Tramadol consumption was recorded between 0 and 1 h, and 1–12, 12–24, a total of 24 h. PONV scores were evaluated using a verbal descriptive scale (0 = none, 1 = mild nausea, 2 = moderate nausea, 3 = vomiting once, 4 = multiple vomiting). The evaluation of the sedation level of the patients was scored on a 4-point scale (0 = awake, 1 = sleepy, easy to wake verbally, 2 = sleepy, 3 = does not open eyes to verbal commands). The severity of nausea was evaluated on a 4-point scale (0 = none 1 = mild, 2 = moderate 3 = severe). PONV and sedation scores were recorded at each time point of NRS evaluations.

The demographic characteristics of the patients were recorded before the surgery, and their first oral intake time and unaided standing time were recorded after the surgery. All the outcome measures were recorded by an anesthesiologist who was blinded to the group allocations.

### Postoperative analgesia and antiemetic use

All patients were routinely administered iv 1 g paracetamol every 8 h (the first dose was administered before surgical incision with tenoxicam 20 mg IV) If the patient’s NRS score was 4 or above, iv 50 mg tramadol was administered as rescue analgesia within 2–3 min. If the nausea score of the patient was ≥ 2, the patient was administered iv 10 mg metoclopramide.

### Statistical analysis

The statistical analyses of the study were performed using the JASP package program. The descriptive statistics of the quantitative variables of the study are shown as arithmetic means, standard deviation, median, minimum and maximum values, and the qualitative variables are shown as frequency and percentage. The conformity of the quantitative variables to normal distribution was examined using the Shapiro-Wilk test. The normal Mann-Whitney U test was used in two independent group comparisons of non-normally distributed variables. Pearson’s Chi-square test was used in independent group comparisons of qualitative variables. The changes in quantitative variables with respect to time were evaluated using the Friedman test. Results below 0.05 were considered statistically significant in all statistical analyses.

A reduction in the NRS ≥ 2 was considered clinically meaningful with a standard deviation of 1.78. [11] At least 31 patients were required for each group for a power of 90% with a two-sided significance level of 5% [11]. We included 34 patients per group considering a possible drop-out rate of 10%.

## Results

M-TAPA was successfully performed on all patients randomized to the block group, uneventfully. All patients were routinely observed for 1 h in the recovery room.

Figure 1 shows the CONSORT diagram of enrollment for this study. After the exclusion of three patients (history of abdominal surgery n = 1, BMI ≥ 35 kg/m^2^ n = 2), 68 patients were included in the randomization process. Demographic data are shown in Table 1. There was no statistically significant difference between the two groups with regards to these parameters. The first oral intake times of those in group M-TAPA were found to be significantly earlier (P = 0.028).

**Table 1:** Demographic Data

	Group M-TAPA (n = 34)	Group Control (n = 34)	P value	Mean/ Median difference (95% CI)
Age (years)	49.5 (26–74)	50 (21–68 )	0.400^#^	-0.5 (-5–17)
Gender(F/M)	22/12	25/9	0.600^#^	
Weight (kg)	80 (70–90)	75 (45–105)	0.169^#^	
Height (cm)	165 (155–185)	164.5 (152–180)	0.702^#^	0.5 (-1–5)
BMI (kg/m^2)^	28.3 ± 2.52	27.33 ± 3.69	0.221*	
ASA	2 (1–2)	2 (1–2)	0.760^#^	0 (0–1)
Oral intake time (hour)	7 (4–20)	8 (6–26)	0.028^#^	-1 (-3–0)
First mobilization time (hour)	5 (2–13)	5 (2–13)	0.285^#^	0 (1–3)
Anesthesia time (minute)	53.50 (35–100)	50 (25–120)	0.095^#^	3.5 (0–15)
Surgical time (minute)	45 (28–90)	41 (20–110)	0.150^#^	4 (5–20)

BMI: Body mass Index, ASA: American Society of Anesthesiologists physical status, M-TAPA: modified-thoracoabdominal nerves block through a perichondrial approach, CI: confidence interval (^#^Mann Wihtney U test, *Independent Samples t test. Values are presented as median (minimum, maximum)

**Fig. 3:**
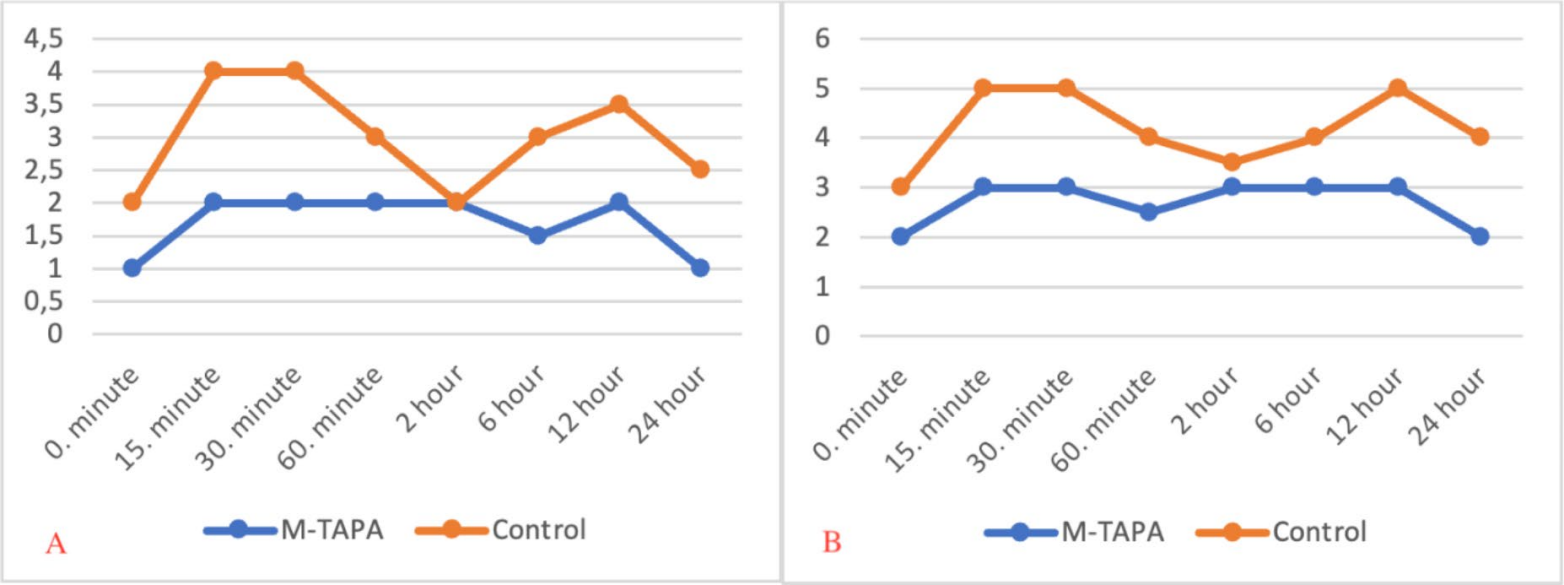
**NRS change between the 15th minute and 24 h at rest and movement** **(A)** Average NRS scores at rest, for Group M-TAPA and Group Control at various time points of follow-up. **(B)** Average NRS scores at motion, for Group M-TAPA and Group Control at various time points of follow-up. M-TAPA: modified-thoracoabdominal nerves block through a perichondrial approach, NRS: numerical rating scale

NRS values were statistically significantly lower in group M-TAPA than in the group control, both during resting and motion from the 15th minute to the 24th hour (P < 0.01 at each time point) (Table 2). In group M-TAPA and the group control, the NRS change between the 15th minute and 24 h at rest and at movement was statistically significant (P < 0.05) (Fig. 3) (#Friedman test).

**Table 2:** Median NRS scores and minimum-maximum values of the groups at different time points

Time frame	Group M-TAPA	Group Control	P value
*0th minute*			
Resting	1 (0–5)	2 (0–6)	**0.005** ^**#**^
Motion	2 (0–6)	3 (1–8)	**< 0.001** ^**#**^
*15th minute*			
Resting	2 (0–5)	4 (1–7)	**< 0.001** ^**#**^
Motion	3 (0–6)	5 (2–8)	**< 0.001** ^**#**^
*30th minute*			
Resting	2 (0–5)	4 (2–6)	**0.001** ^**#**^
Motion	3 (1–7)	5 (3–8)	**< 0.001** ^**#**^
*1st hour*			
Resting	2 (0–4)	3 (1–6)	**< 0.001** ^**#**^
Motion	2.5 (1–5)	4 (3–8)	**< 0.001** ^**#**^
*2nd hour*			
Resting	2 (0–5)	2 (1–6)	**< 0.001** ^**#**^
Motion	3 (1–6)	3.5 (2–6)	**0.002** ^**#**^
*6th hour*			
Resting	1.5 (0–5)	3 (1–5)	**< 0.001** ^**#**^
Motion	3 (1–6)	4 (2–7)	**< 0.001** ^**#**^
*12th hour*			
Resting	2 (0–4)	3.5 (1–5)	**< 0.001** ^**#**^
Motion	3 (1–6)	5 (2–7)	**< 0.001** ^**#**^
*24th hour*			
Resting	1 (0–4)	2.5 (0–6)	**< 0.001** ^#^
Motion	2 (0–5)	4 (2–8)	**< 0.001** ^#^

Statistically significant differences are highlighted in bold. ^#^Mann Whitney U test. (M-TAPA: modified-thoracoabdominal nerves block through a perichondrial approach; Numerical rating scale: NRS)

Tramadol was required in 21 patients in group M-TAPA and 34 patients in the group control in the recovery room, during the 1st postoperative hour (P < 0.01). From the 1st hour to the 12th hour, rescue analgesia was required in 15 patients in group M-TAPA and 26 patients in the group control (P = 0.002). In the second 12 h, rescue analgesia was required in 14 patients in group M-TAPA and 31 patients in the group control (P < 0.001). The number of patients requiring rescue analgesia was significantly lower in group M-TAPA at all time intervals (P < 0.01 for 0–1 h; P = 0.002 for 1–12 h; P < 0.001 for 12–24 h).

Postoperative tramadol requirement was significantly lower in group M-TAPA than in the group control in the first 24 h (100 [0-200] mg vs. 200 [100–300] mg, P < 0.001) (Table 3).

**Table 3:** Comparison of postoperative analgesia requirement between groups for first 24 h

Time Frame	Group M-TAPA	Group Control	P value
Tramadol(mg) 0–1 (first h)	50 (0–100)	100 (50–200)	**< 0.001** ^**#**^
Tramadol(mg)0–12 (first12 h)	0 (0–100)	50 (0–200)	**0.002** ^**#**^
Tramadol(mg)12–24(second12h)	0 (0–100)	50 (0–150)	**< 0.001** ^**#**^
Tramadol(mg) 0–24 h (first 24 h)	100 (0–200)	200 (100–300)	**< 0.001** ^**#**^

Statistically significant differences are highlighted in bold. ^#^Mann Whitney U test. Values are presented as median (minimum, maximum). (M-TAPA: modified-thoracoabdominal nerves block through a perichondrial approach)

The preoperative and postoperative mean (median difference) global and dimensional QoR-40 scores are presented in Table 4. The median (minimum-maximum) postoperative global QoR-40 scores were significantly better in group M-TAPA than in the group control (189 [156–198] vs. 179.5 [146–191] (P < 0.001) (Table 4).

**Table 4:** Total and Dimensional QoR-40 Scores of the Participants

	Group M-TAPA (n = 34)	Group Control (n = 34)	P value	Mean/ Median difference (95% CI)
*Preoperative*				
Physical comfort	56 (46–59)	56.5 (42–60)	0.541^#^	-0.5 (-3–2)
Emotional status	42 (35–45)	42 (31–45)	0.660^#^	0 (-1–2)
Physical independence	25 (15–25)	25 (18–25)	0.312^#^	0 (0–3)
Psychological support	35 (33–35)	35 (33–55)	0.274^#^	0 (0–1)
Pain	32 (24–35)	31.5 (25–35)	0.762^#^	0.5 (-1–1)
Global QoR40	188.5 (168–198)	189 (163–198)	0.773^#^	-0.5 (-3–4)
*Postoperative*				
Physical comfort	55 (39–60)	51 (39–60)	**0.001** ^#^	4 (1–5)
Emotional status	43.5 (35–45)	41 (32–45)	**0.001** ^#^	2.5 (0–4)
Physical independence	22.5 (14–25)	22 (17–25)	**0.005** ^#^	0.5 (0–3)
Psychological support	35 (34–35)	35 (32–35)	0.247^#^	0 (0–2)
Pain	33 (26–35)	29.5 (22–34)	**< 0.001** ^#^	3.5 (2–3)
Global QoR40	189 (156–198)	179.5 (146–191)	**< 0.001** ^#^	9.5 (4–14)

Statistically significant differences are highlighted in bold and italic. ^#^Mann Whitney U test. Values are presented as median (minimum, maximum). (M-TAPA: modified-thoracoabdominal nerves block through a perichondrial approach, QoR40: Quality of Recovery- 40 score, CI: Confidence Interval)

No statistically significant differences (P > 0.05) were observed between the study groups in the sedation score, except at the 60th minute. The PONV scores of both groups were similar (P > 0.05), except for the 15th minute. There were no differences in metoclopramide consumption at any time point.

## Discussion

The present study showed that ultrasound-guided bilateral M-TAPA block after the induction of general anesthesia reduced NRS scores significantly at all time points in patients undergoing LC when compared with the control group and resulted in less opioid requirement in the first 24 h. In addition, M-TAPA block improved the quality of recovery in these patients.

Following LC, the largest component (50–70%) of the total abdominal pain after surgery originates from the incision sites, followed by pneumoperitoneum (20–30%), and cholecystectomy (10–20%) [12]. The mechanisms that trigger postoperative pain are multifactorial, suggesting that it is difficult to control postoperative pain after LC. For this reason, multimodal analgesia is preferred. Multimodal analgesia methods, which include peripheral blocks, decrease the consumption of analgesics and related adverse effects [13]. Among these techniques, neuraxial blocks including thoracic epidural blocks have been accepted as technically difficult and the incidence of intervention-related complications is also high [14]. The use of ultrasound-guided interfascial plane blocks, which are considered to be easy and safe, has recently increased in LC surgery. Recent studies evaluated the effects of OSTAP, transversus abdominis plane (TAP), ESP, and paravertebral blocks on postoperative analgesia in LC surgery [6, 11, 15].

OSTAP block performed to provide postoperative analgesia after upper abdominal surgery cannot block lateral cutaneous branches of the intercostal nerves effectively [16, 17]. The study of Borglum et al., suggested dual TAP for blockage of the upper (T6-9) and lower TAP plexus (T10-12) [18].In a previous study, it was shown that TAP block included T10-L1 nerves and such a spread did not provide the required sensory blockade on the incision site in LC surgery [19]. In the present study, the M-TAPA block was preferred because it was speculated to provide extensive analgesia in the anterior and lateral abdominal regions between T5 and T12 [9, 10].

Although ESP block has recently gained popularity, there is still controversy about its mechanism, and the risk of a patchy blockade is well known [20]. Studies were conducted showing that ESP block brought effective analgesia by blocking visceral and parietal components of the LC, but ESP block could only be performed in prone, lateral, or sitting positions [6, 11].

Paravertebral block is another regional anesthesia technique that can be used in LC [21]. However, it may carry risks of pleural puncture, pneumothorax, epidural invasion, and injection into the subarachnoid space beyond technical difficulty [21]. Again, position-related problems are also valid for this block. M-TAPA block, on the other hand, seems to be a comfortable block for patients and anesthetists and can provide an analgesic effect without taking such risks related to positional changes.

Although port-site infiltration that is another popular analgesic method, seems to create analgesia by blocking somatic nerve fibers, the duration of analgesia after port-site infiltration lasts only 2–3 h compared with interfascial plane blocks in which the duration of analgesia may extend up to 36 h, possibly due to slow clearance of local anesthetics from relatively lack of blood circulation [22]. We think that the long effect of the local anesthetic used in group M-TAPA in the postoperative period was because of the decrease in absorption in an environment with low vascularity [23]. On the contrary, there are opposite opinions postulating that interfascial plane blocks may lead to increase systemic absorption of local anesthetics. El-Boghdadly et al. reported that the time to reach maximum plasma concentration of local anesthetic was different in different interfascial plane blocks. This difference of absorption may also explain the difference of block analgesia time in the postoperative period between different types of interfascial plane blocks [24].

With the perichondrial approach, a thoracoabdominal nerve block is a novel analgesic technique with a broad analgesic effect that involves the injection of LA in both the lower and upper part of the chondrium at the costochondral corner affecting both the anterior and lateral branches of the thoracoabdominal nerves from T5-6 to T11-12 [8]. In the present study, the modified TAPA (M-TAPA) technique, which was performed by injecting the LA as defined by Tulgar et al., only in the lower part of the chondrium, was used [9]. The LA administered under the 10th costal cartilage leads to blockade of both anterior and lateral cutaneous branches of intercostal nerves possibly by passing the space between the costal cartilage and the origin of transversus abdominis muscle without facing the obstruction by linea semilunaris. It was reported that a multi-level intercostal nerve block could be achieved with one single LA injection into the endothoracic fascial plane just below the costas [25]. TAPA block was shown to provide effective postoperative analgesia in patients undergoing LC in a recently published mini-case series [26]. In different case series in which M-TAPA blocks were performed, blockade in the Th7-Th11, and Th3-Th12 range was observed [9, 27]. In Tanaka’s study, analgesic effects ranging from Th6 to Th12 were observed in some cases [28]. However, in the two cadavers evaluated in the same study, spread between T8-11 was observed [28]. It has been stated that this wide range of analgesics may be due to various factors such as pneumoperitoneum, increased intra-abdominal pressure, the retractor effect, and intraoperative position in living humans [28].

There are no previous studies conducted with M-TAPA block in abdominal surgeries comparing the ideal LA concentration and volume. Twenty, 25, and 30-mL volumes of 0.25% LA were used in case reports and single-group studies [8, 9, 26, 27]. In the present study, 25 mL of 0.25% bupivacaine was used on each side. We designed our research as bilateral M-TAPA administration on patients to exclude periumbilical pain during the postoperative period.

Similar to OSTAP and subcostal TAP block, we think that M-TAPA block has a low risk of serious complications with the increased safety with ultrasound guidance. Abdominal wall hematoma, vascular injury, and LA toxicity are rare but potential complications of TAP block [29]. Although the location of the M-TAPA block is close to the lungs, there are no reported complications. In the present study, no block-related complications were observed. We observed no symptoms of local anesthetic toxicity in any patients. Future studies should focus on the time of maximum plasma concentrations of local anesthetics observed after M- TAPA block applications.

Ultrasound-guided M-TAPA block significantly improved the quality of recovery of patients after LC in the present study. All subscales of QoR-40 except for the psychological support subscales were higher in the M-TAPA group at the postoperative 24th hour than in the control group. This result suggests that ultrasound-guided M-TAPA block accelerates recovery after general anesthesia in LC surgery. We believe that the most likely reason for this is the opioid protective effect of M-TAPA block.

The present study had some limitations. To determine the effectiveness of M-TAPA block in multimodal analgesia, the sample size was calculated by considering its effect on postoperative pain scores. However, with the current sample size, there was not sufficient power to analyze less common adverse effects such as PONV. The sensory dermatome that was affected by the M-TAPA block was not identified because this might impair the blinding status of the study.

Another issue that we considered as a limitation in our study is that studies evaluating regional anesthesia techniques use usually morphine for postoperative analgesia. However, tramadol has proved to be an effective analgesic in the treatment of moderate-to-severe acute postoperative pain in adults and has a lower adverse effect profile [30]. Tramadol has a faster onset of action than morphine and was shown to have the same effect as morphine in patients undergoing LC [31].

### Conclusion

Bilateral ultrasound-guided M-TAPA block provides effective analgesia and decreased opioid requirement in patients undergoing LC surgery. It also has positive effects on the quality of recovery. However, comparison trials are still needed regarding its prospective dose and concentration with other regional anesthesia techniques. Although it is premature to recommend M-TAPA for routine use in LC surgery, it can be used as an effective technique.

## Data Availability

The datasets used and/or analyzed during the current study are available from the corresponding author on reasonable request.

## Declarations

### Clinical trial registration

The study was registered prospectively at clinicaltrials.gov (trial ID: NCT05017090 on 23/08/2021)

## List of abbreviations

M-TAPA: Modified thoracoabdominal nerve block through the perichondrial approach
LC: Laparoscopic cholecystectomy
PONV: Postoperative nausea and vomiting
QoR-40: Quality of Recovery-40
OSTAP: Oblique subcostal transversus abdominis plane block
SIP: Serratus intercostal plane block
ESP: Erector spinae plane
TAPA: Thoracoabdominal nerves through perichondrial approach
ASA: American Society Anesthesiologists
LA: Local anesthetic
CONSORT: Consolidated Standards of Reporting Trials
BMI: Body mass index
PACU: post-anesthesia care unit
NRS: Numerical rating scale
TAP: Transversus abdominis plane
